# The Effect of Glyphosate on the Reproduction of Estuarine Crabs: *Neohelice granulata* as a Study Model

**DOI:** 10.3389/fendo.2021.643168

**Published:** 2021-03-26

**Authors:** Enrique M. Rodríguez, Daniel A. Medesani, Ivana S. Canosa, Luciana Avigliano

**Affiliations:** ^1^ Laboratorio de Fisiología de Crustáceos, Universidad de Buenos Aires, CONICET, Instituto de Biodiversidad y Biología Experimental y Aplicada (IBBEA), Facultad de Ciencias Exactas y Naturales, Departamento de Biodiversidad y Biología Experimental, Ciudad Universitaria, Buenos Aires, Argentina; ^2^ Instituto del Conurbano—Universidad Nacional de General Sarmiento (ICO-UNGS), CONICET, Los Polvorines, Argentina

**Keywords:** Roundup Ultramax^®^, crabs, ovary, herbicide, reproductive aspects, spermatozoa

## Abstract

This review summarizes the bulk of evidence about the effect of glyphosate, both technical and formulated, on the ovarian maturation of *Neohelice granulata* female crabs, as well as the effects of glyphosate on sperm production in males of the same species. After long-term *in vivo* assays, made during the 3-month pre-reproductive period of this species, both formulated and technical glyphosate were able to produce a significant incidence of oocyte reabsorption in the ovary, together with a concomitant decreased of vitellogenin content, at concentrations ranging from 0.2 to 1 mg/L. Despite this, after 32-day *in vivo* assays, glyphosate stimulated oocyte growth, in terms of a higher percentage of vitellogenic oocytes, suggesting that glyphosate could be acting as an endocrine disruptor. *In vitro* assays made with isolated ovarian pieces showed a decrease of vitellogenin content, in correlation with lower protein synthesis, although some advance in maturation was observed in the histological analysis. In male crabs exposed *in vivo* to both technical and formulated glyphosate at 1 mg/L, several reproductive imbalances were noted, such as a significant decrease of the sperm count, abnormal spermatophores, and possible disrupting effects of glyphosate on the androgenic gland.

## Introduction

Glyphosate [N-(phosphonomethyl) glycine] is currently the most widely used herbicide worldwide (1). This is a non-selective, systemic herbicide that inhibits the enzyme 5-enolpyruvylshikimate-3-phosphate synthase, involved in the aromatic amino acid synthesis in plants (2). In the last two decades, the use of glyphosate in Argentina has increased exponentially, mainly on transgenic soy resistant to glyphosate (3, 4). Consequently, environmental levels of glyphosate have been reported to range between 0.1 and 0.7 mg/L in water and between 0.5 and 5 mg/kg in sediment (5).

Crustaceans are a highly representative group of the invertebrate aquatic fauna susceptible to be impacted by glyphosate (6). In particular, *Neohelice granulata* (Dana, 1851) is a widespread estuarine crab species, distributed along the Atlantic coast of both Argentina and Brazil. In Argentina, this species forms very dense populations along the entire coast of Samborombon Bay, corresponding to the external zone of the “Rio de la Plata” estuary, also being a relevant link in the trophic web that includes several fish species that reproduce there (7). This Bay, as well as other sites inhabit by *N. granulata*, received the discharge of several rivers and channels that cross extensive agricultural areas and carry significant amounts of pesticides, including herbicides (8). Moreover, glyphosate is more intensively applied to both soybean and corn crops during summer, coinciding with the reproductive period of *N. granulata* (9).

The reproductive cycle of *N. granulata* comprises three periods (9): pre-reproductive (winter), during which the ovary grows and mature; reproductive (spring and summer), when both spawning and hatching occur, and eventually the ovarian re-maturation takes place to produce further spawns; and finally the post-reproductive period (autumn), which leads to both ovarian quiescent and molting of adults. In addition to the production of vitellogenin by the oocytes themselves (primary vitellogenesis), during the pre-reproductive period, the ovary enters into the secondary vitellogenesis, which implies an active uptake by maturing oocytes of the vitellogenin synthesized both in the hepatopancreas and the ovary (10, 11). Vitellogenin is the precursor of vitellins, the main lipoproteins of the yolk. After spawning, hatching, and larval development, the megalopa returns to the coast, molting successively to several juvenile instars, and finally reaching the adult condition (9).

Crustacean vitellogenesis is under hormonal control; to this respect, several neurohormones by the thoracic ganglion, brain, and the X organ–sinus gland complex located at the eyestalks are involved (12). These neurohormones, in turn, regulate the secretion of other hormones of non-peptidic nature; one of them is represented by vertebrate-like steroids such as 17-hydroxyprogesterone, likely secreted by the ovaries (11, 13); moreover, progesterone receptors have been identified in the ovary and other tissues of shrimps and crabs (14, 15). Besides, methyl farnesoate, the juvenile crustacean hormone secreted by the mandibular organ, as well as prostaglandins, have been reported as other stimulating hormones of crustacean reproduction (12, 16, 17). In males, the endocrine regulation of spermatogenesis is mainly carried out by the androgenic gland, a bilateral organ attached to the distal region of the *vasa deferentia* (18), which secretes the “androgenic gland hormone”, belonging to the insulin family, under the control of the neurohormones secreted by both eyestalks and thoracic ganglion (11, 19).

Relatively few studies on the deleterious effect of glyphosate on crustacean reproduction have been published, in comparison with the literature available for other aquatic species, such as fishes. Concerning the role of glyphosate as a possible endocrine disruptor, some evidence on the interference of this herbicide with the synthesis of several sexual steroids has been also reported in animal models other than crustaceans (1, 20, 21). This review is aimed at summarizing the deleterious effects of glyphosate, both technical and formulated, on the reproduction of the crab *N. granulata*, taken as a representative model of higher crustaceans. For this, the results of both *in vivo* and *in vitro* assays are presented. Among the imbalances caused by glyphosate, several pieces of evidence about the endocrine disruption exerted by this herbicide are discussed, in both females and males.

## *In Vivo* Effects on Female Crabs

**Figure 1:**
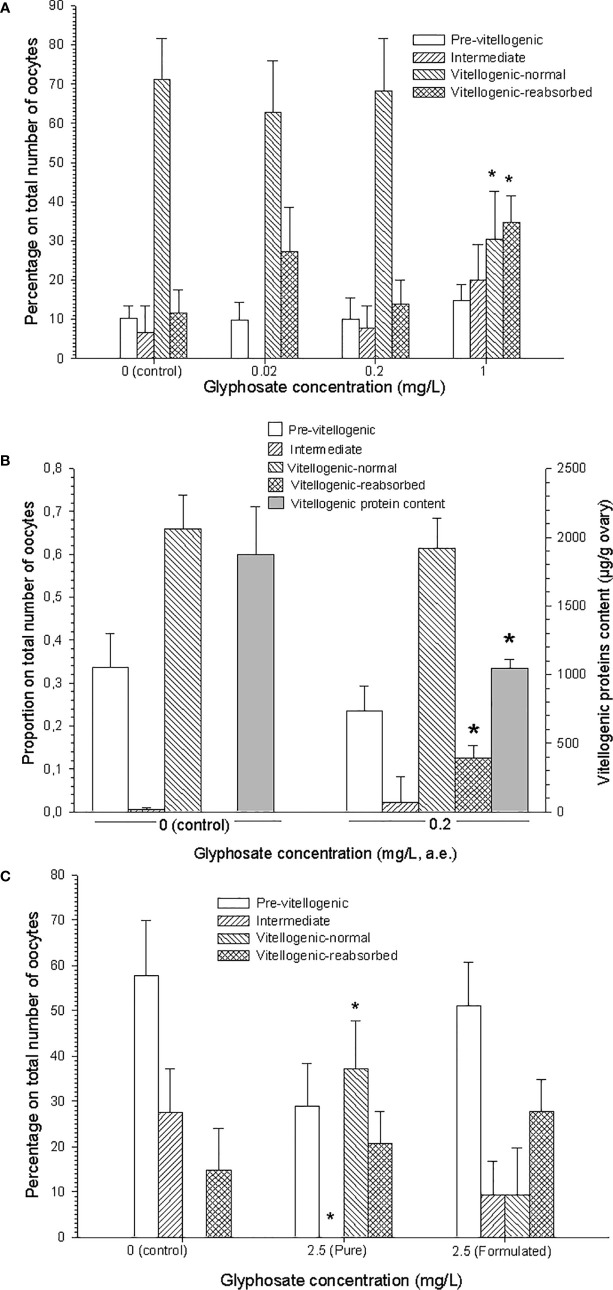
Results from *in vivo* assays made with glyphosate on the crab *Neohelice granulata.* **(A)** Assay made with technical glyphosate during the entire (3-months) pre-reproductive period. Reproduced from Avigliano et al. (22). **(B)** Assay made with Roundup Ultramax^®^ during the same period. Adapted from Canosa et al. (23). **(C)** Assay made with both technical and formulated (Roundup Ultramax^®^) glyphosate, during 32 days, in post-hatching females. Redrawn from Avigliano et al. (24, 25). In all cases, glyphosate concentrations are expressed as acid equivalents (a.e.); oocyte types are indicated; asterisks indicate significant differences (p < 0.05) with respect to control. All figures are reproduced or adapted with permission.

**Figure 2:**
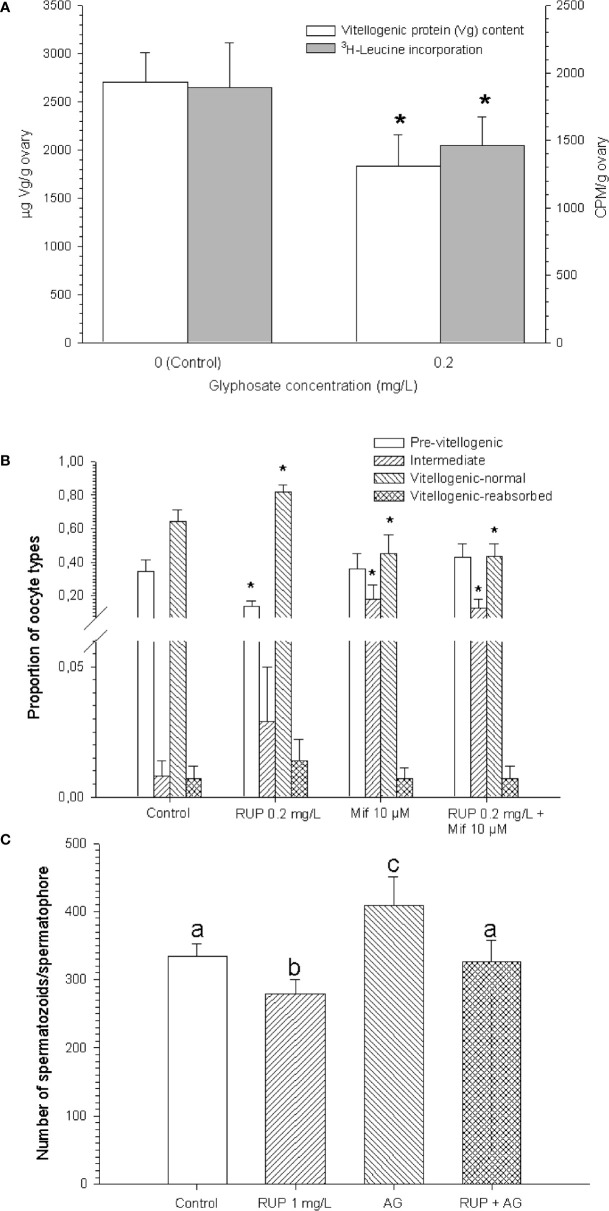
Results from *in vitro* assays made with formulated glyphosate (RUP: Roundup Ultramax^®^) on the crab *Neohelice granulata*. **(A)** Vitellogenic protein (Vg) content, and ^3^H-leucine incorporation. Adapted from Canosa et al. (23). **(B)** Histological analysis; oocyte types are indicted; Mif, mifepristone. Redrawn from Canosa et al. (26). **(C)** Sperm count in males; AG, androgenic gland. Reproduced from Canosa et al. (27, 28). In all cases, glyphosate concentrations are expressed as acid equivalents (a.e.); asterisks, as well as different lowercase letters, indicate signficant differences (p < 0.05) with respect to control. All figures are reproduced or adapted with permission.

A first *in vivo* assay carried out with technical glyphosate during the entire pre-reproductive period (3 months), showed a significant incidence of oocyte reabsorption, but just at the highest glyphosate concentration tested [~ 35% in 1 mg/L concentration, Figure 2 in (22), **Figure 1A** here]. A further *in vivo* assay, made during the same period with the commercial glyphosate formulation Roundup Ultramax^®^, also showed a significant incidence of oocyte reabsorption in the ovary, together with decreased vitellogenic protein content by gram of ovary; these results were found in females exposed to 0.2 mg/L of glyphosate presented as the active ingredient in the commercial formulation above mentioned [Figures 2 and 3 in (23), **Figure 1B** here]. Based on the results of these long-term assays, contributive toxicity of the coadjuvants presented in the commercial formulation seems to have been occurred.

A third *in vivo* assay was made during the first part of the reproductive period (spring), with re-maturating females, i.e., females that became ovigerous at the beginning of this period, whose ovaries entered at the same time in a re-maturation phase, in order to produce a second spawn. After exposing post-hatching females for 32 days to both technical and formulated (Roundup Ultramax^®^) glyphosate, a significant advanced oocyte growth was seen with technical glyphosate, in terms of a higher percentage of vitellogenic (more mature) oocytes [Figure 5B-C and Table 2 in (24, 25), **Figure 1C** here]. Besides, oocyte reabsorption was observed in all treatments (although with some higher incidence in the glyphosate treatments than in the control), because the remnant of oocytes not released in the previous spawn was under reabsorption in all females.

## *In Vitro* Effects on Female Crabs

*In vitro* assays comprised the incubation of isolated ovarian pieces for 24 hours, in small vials with an appropriate culture medium, and into a culture chamber. After incubating isolated pieces of ovary with Roundup Ultramax^®^, at the same glyphosate concentration that showed deleterious effects *in vivo* (0.2 mg/L), a decrease in vitellogenin content was also seen. Moreover, by means of evaluating the incorporation of a radiolabeled amino acid to the ovarian proteins, it could be established that partial inhibition of the protein synthesis was caused by Roundup Ultramax^®^ [Figure 6 in (23), **Figure 2A** here], accounting for the decreased vitellogenin content observed *in vitro*. Short-term changes in vitellogenic protein content (either increase or decrease) have been also observed by effect of other pollutants, and even hormones, in several *in vitro* assays made on *N. granulata* and other crustacean species (24, 25, 29–32). The vitellogenin synthesis can certainly rapidly detected; as reported by Reddy Buchi et al. (33), the expression of vitellogenin ARNm occurred as early as 3 hours after beginning the *in vitro* incubation of crab hepatopancreas.

Another *in vitro* assay made with technical glyphosate at 0.2 mg/L showed an abnormal increase in the size of the immature (previtellogenic) oocytes, an effect also observed *in vivo* (22, data not shown). These results suggest that glyphosate could stimulate the secretion of any stimulating hormone produced by the ovary, such as some kind of ovarian steroid (11, 34), or could be acting as a steroidal agonist, by binding to steroid receptors at the ovary. To check this latter hypothesis, a histological analysis was done after a 24h-incubation of ovarian pieces with Roundup Ultramax^®^ (0.2 mg/L), mifepristone 10 µM (an antagonist of progesterone receptors), or the combination of Roundup and mifepristone. This antagonist, either alone or in combination with Roundup, inhibited the oocyte’s maturation, although Roundup alone was able to stimulate it [Figure 3 in (26), **Figure 2B** here]. Although Roundup *per se* stimulated the ovarian maturation, in terms of an increased proportion of vitellogenic oocytes, it also reduced the area of these oocytes [Table 1 in (26), data not shown], which was in agreement with the lower vitellogenic protein content observed by the effect of Roundup in the previous *in vitro* assay mentioned.

These later results, together with the advanced re-maturation observed *in vivo* after 32-days of exposure, are consistent with a possible endocrine-disrupter effect caused by glyphosate, likely altering the secretion and/or the transductional mechanism of one or more hormones controlling the ovarian growth in crustaceans.

## Effects on Male Crabs

*Neohelice granulata* male crabs exposed for 30 d to 1 mg/L of either technical or formulated glyphosate, showed a significant decrease in weight gain and muscle protein levels, a result previously observed in juveniles of the crayfish *Cherax quadricarinatus* (35–37). Besides, in spermatophores from the *vasa deferentia* of those *N. granulata* males, a significant decrease of the sperm count was observed by effect of Roundup Ultramax^®^, while a significant incidence of abnormal spermatophores was observed either with technical glyphosate or Roundup treatment (27, 28). Moreover, after incubating testes and *vasa deferentia* with Roundup Ultramax^®^, with or without the presence of the androgenic gland, the increase in the number of spermatozoa/spermatophore produced by the co-incubation with this endocrine gland was completely reverted by the addition of Roundup [Figure 5 in (27, 28), **Figure 2C** here].

## Discussion and Conclusions

The concentrations used in the *in vivo* assays were within the environmental range of glyphosate reported for several water bodies of Argentina (5, 38). The deleterious effects on the ovarian growth were observed at 0.2 mg/L of glyphosate (as active ingredient in Roundup Ultramax), a concentration lower than the 0.7 mg/L reported for argentine most impacted areas (5). Besides, glyphosate application in Argentina has been steadily increasing over time (3); more than 20 million ha (mainly soybean crops) are currently treated with approximately 200 million liters of glyphosate (5). Since glyphosate has not shown to be acutely toxic for aquatic fauna, its chronic accumulative effects represent a critical issue to be studied. Certainly, this has been the focus of our *in vivo* studies on the estuarine crab *N. granulata*, during the entire period of ovarian growth. Besides, since several undeclared surfactants have been used during the last years to formulate Roundup and other trademarks (1), the study of the deleterious effects caused by technical glyphosate represents useful information for evaluating the toxicity of any commercial formulation of this herbicide.

The decrease in vitellogenin content observed *in vitro* with 0.2 mg/L of glyphosate (in the Roundup Ultramax^®^ formulation), was related to an inhibitory effect of the herbicide on the vitellogenic protein synthesis, as also observed *in vitro*. Moreover, the results of the 90-days *in vivo* assays, either made with Roundup Ultramax^®^ at 0.2 mg/L or with technical glyphosate at 1 mg/L, showed significant oocyte reabsorption. Reactional oocyte atresia has been observed in previous studies made on *N. granulata* females exposed to several pollutants (39–42). In this kind of atresia, follicular cells that surround the vitellogenic oocytes invade them to phagocyte the vitellum. Interestingly, atretic oocytes have also been reported in several frog species chronically exposed to either 0.6 or 1.8 mg/L (a.e.) of the original Roundup^®^ formulation (43).

Within an *in vivo* context, the reabsorption of yolk could be a compensatory mechanism when a reduction in somatic growth is taking place as a result of stressful situations (44). In fact, in those females exposed to technical glyphosate for three months, a decrease in the body weight gain was observed at all concentrations assayed (22). In addition, early juveniles of the crayfish *C. quadricarinatus* exposed to technical glyphosate (10 and 40 mg/L) also showed a decreased body weight gain, in correlation with lower levels of both protein and lipid reserves (36, 37). Advanced juveniles of the same species also showed a growth reduction after being exposed to 15 mg/L of a mixture of glyphosate with polyoxyethylenamine, the surfactant used in the original formulation of Roundup^®^ (35). This formulation was also able to reduce the growth rate of freshwater shrimps exposed for 40 days to concentrations ranging from 2.2 to 5.4 mg/L (36, 37). Another glyphosate formulation inhibited the growth of the first copepodite stage, preventing copepods to reach the adult stage, these effects being observed at a glyphosate concentration of 0.81 mg/L (27, 28).

Some hormones belonging to the CHH-family of peptides, produced and secreted at the eyestalks, could be involved in oocyte reabsorption. The CHH (crustacean hyperglycemic hormone) has been considered as the “stress hormone” of crustaceans (45), and some effects on the ovary of different isoforms of CHH have been also reported in several crustacean species (46, 47). Another hormone of this family, the GIH (gonad inhibiting hormone) would prevent the maturation of pre-vitellogenic oocytes to vitellogenic ones (12). If the titers of this inhibitory hormone were high when the ovary is maturating, it is plausible to suppose that vitellogenic oocytes could be suffering reabsorption. Interestingly, glyphosate was able to increase oocyte reabsorption when eyestalk tissue was added to ovarian pieces *in vitro* (22, data not shown).

However, an advanced ovarian maturation, in terms of a higher proportion of vitellogenic oocytes, was seen after the 32 days-long *in vivo* assay with re-maturating females. Nevertheless, by comparing this result with the inhibition of ovarian growth observed after the long-term (90 days) *in vivo* assay, the stimulating effect observed after 32 days seems to have been overcome after 90 days of exposure, when the oocyte reabsorption and the consequent decrease in the vitellogenic protein content were evident, as compared to control. Stimulation of ovarian growth has been also seen in *N. granulata* females exposed to the organophosphate insecticide parathion, in terms of a larger size of both previtellogenic and vitellogenic oocytes (39). Indeed, the abnormal acceleration of ovarian growth observed with either glyphosate or parathion strongly suggests that these pesticides are able to act as endocrine disruptors in crustaceans.

In this respect, an interference with the endocrine control of ovarian maturation exerted by steroids could be a serious possibility to be considered, and in this sense, the results from the *in vitro* assay with mifepristone suggest that glyphosate could act as a “xenoprogestagen”, facilitating the transductional pathway of progesterone at some step, or eventually enhancing the synthesis of progesterone by the ovary, in order to produce a higher proportion of vitellogenic oocyte. Progesterone, among other steroids, has been suggested to play different roles in the ovarian maturation of crustaceans, other than that strictly related to the synthesis of vitellogenic proteins (34). Taken together, the results from the *in vitro* assays indicate that glyphosate could exert a direct effect on the ovary by inhibiting the synthesis of vitellogenic proteins to some extent, as mentioned earlier, but at the same time it could produce an advanced maturation by potentiating the effect of some steroid hormones secreted by the ovary, such as progesterone. However, the exact mechanism by which glyphosate and/or the other components of the glyphosate formulation assayed would directly affect the maturation process in the ovary needs further research. Besides, the extrapolation of the results obtained *in vitro* to the *in vivo* conditions should be carefully made, since the *in vivo* physiological context is certainly more complex, and therefore a lot of interacting effects should be taking into account.

The endocrine control of reproduction in crustacean males has been relatively less studied than in females. However, it is well known, as mentioned previously, that the androgenic glands attached to the *vasa deferentia* are responsible for secreting a peptidic hormone that regulates sexual differentiation and spermatogenesis (11, 19). The *in vitro* suppression caused by Roundup Ultramax^®^ on the increased number of spermatozoa stimulated by the androgenic gland, suggests that inhibition of the secretion and/or transduction of the androgenic gland hormone could have been caused by this glyphosate formulation, in addition to some other deleterious effects caused by the herbicide, such as abnormal spermatophores. Comparatively, fish exposed to 3.6 mg/L of the original Roundup^®^ formulation, showed a reduction in sperm quality (48).

Evidence about de-masculinization of crustacean males has been reported for some anthropogenic agents. For instance, a lower proportion of males, together with a higher incidence of intersex, were seen in amphipods exposed to industrial effluents in natural environments (49). Moreover, Mac Loughlin et al. (50) have reported a decrease in the proportion of males in early juveniles of the crayfish *C. quadricarinatus* exposed to the herbicide atrazine during the critical period of sexual differentiation. As seen on *N. granulata* exposed both *in vivo and in vitro* to glyphosate, a possible interference of several other pollutants with the endocrine control exerted by the androgenic gland is quite possible.

In summary, taking into account the whole evidence obtained from the estuarine crab *N. granulata* exposed to glyphosate, both technical and formulated, we can conclude the following:

- Glyphosate is able to inhibit the ovarian growth of *N. granulata* females during the 90-days needed for fully ovarian growth, producing a higher incidence of oocyte reabsorption together with decreased vitellogenic protein content. Since these effects were observed at 0.2 mg/L of glyphosate in the Roundup Ultramax^®^ formulation, and just at 1 mg/L of technical glyphosate, the contributive toxicity of the coadjuvants presented in the commercial formulation was evident.
- At a shorter time of exposure (32 days) glyphosate is able to produce an advanced maturation, but only in terms of a higher proportion of vitellogenic oocytes.
- The results of the short-term *in vitro* assays with isolated ovarian pieces indicate an inhibitory effect of glyphosate on the synthesis of vitellogenic proteins, but also a stimulating effect on the progress of maturation, in terms of a higher proportion of vitellogenic oocytes, likely by enhancing the effect of ovarian steroids such as progesterone.

## Author Contributions

ER: supervision of all the studies presented and wrote the manuscript. DM: coordination and supervision of all the *in vitro* experiments and collaboration in the *in vivo* experiments. IC and LA: conduction of experiments and also a PhD student. All authors contributed to the article and approved the submitted version.

## Funding

The experimental work on *N. granulata* referred to in the present review, was supported by grants from ANPCyT (PICT2010-0908, and PICT2016-0040), CONICET (PIP2010-0908, code 100884, and PIP2015, code 11220150100100CO), and the University of Buenos Aires (UBACYT 2016–2018, code 20020150100060BA, and UBACYT 2020, code 20020190100014BA).

## Conflict of Interest

The authors declare that the research was conducted in the absence of any commercial or financial relationships that could be construed as a potential conflict of interest.
